# Engaging with uncertainty when engagement is uncertain

**DOI:** 10.3389/fbinf.2026.1801017

**Published:** 2026-07-09

**Authors:** Nuria Altimir

**Affiliations:** Toiminimi Nuria Altimir, Turku, Finland

**Keywords:** complexity, data visualization, literacy, signal-suppression, uncertainty

## Abstract

Uncertainty is intrinsic to scientific measurement, modelling, and forecasting, yet data visualization practices typically frame uncertainty communication as a task-oriented problem requiring statistical literacy. As a result, uncertainty is rarely leveraged as a resource for engagement, curiosity, or learning, especially for non-expert and non-motivated audiences. This paper examines uncertainty communication through the lens of data visualization design, drawing on cognitive psychology and aesthetic theory. Intuitive, gist-based encodings can reduce cognitive load, support pre-attentive processing, and foster reflective engagement. Adopting such encodings more broadly could advance uncertainty literacy and enable visualizations that are both epistemically rigorous and experientially engaging. Achieving this requires closer integration between statistical thinking and design practice.

## 1 Introduction

All measurements, projections, forecasts, and scientific statements that scientific activity uses or produces carry uncertainty. Samples capture only fragments of reality, instruments have limited accuracy and precision, models rest on assumptions and parameterizations, and forecasts accumulate the compound uncertainty of all preceding steps (both technical and epistemic). We need to calculate uncertainty in science to determine the reliability of a measurement, enable valid comparisons between results, and ensure that data is used to make sound decisions. Uncertainty is intrinsic to scientific work and shapes the meaning of its outputs, so understanding it is essential to interpreting scientific results (Fischhoff, 2012).

This has two practical consequences: uncertainty must be communicated as part of knowledge transfer, and recipients must be able to interpret that information.

Research on uncertainty communication has explored and debated both aspects at multiple levels. The common point of view has been to design strategies that match the user’s capacity to the communication purpose; a purpose that is commonly framed in terms of task performance and decision making of a readily interested audience. For communication goals led by engagement and inspiration, uncertainty is rarely considered as conduit to generate interest.

This piece examines these ideas in the context of data visualization.

## 2 Is anyone uncertainty literate?

The central debate about communicating data uncertainty is driven by concerns about the literacy of the receiver.

The statistical and epistemically sophistication behind uncertainty estimations are complex. At the very technical, the evaluation and expression of uncertainty need the support of formal guidelines that center on the numerical calculation (JCGM, 2011; JCGM, 2008; JCGM, 2023). The field is wide and there are several techniques to approach uncertainty evaluation (Cuzzolin, 2025). Mismatched literacy between experts is an issue, rooted in technical approaches that serve different disciplines. This brings up the need for harmonization between disciplines in multidisciplinary studies, like IPCC (Frey et al., 2006), to discuss communication supporting decision-making strategies (Dhungana et al., 2025; Fischhoff and Davis, 2014), and publish guidelines, recommendations and revision addressing specific professional ambits (EFSA et al., 2018; Mastrandrea et al., 2010; OSR, 2022; Yanai et al., 2021).

Literacy is widely discussed for the communication of data uncertainty to non-experts, from whom we can hardly expect such nuanced and niche level of understanding that not even experts can manage. Discussions include the role of media, and the effect that uncertainty communication has on science trust from the general public. Research shows that technical and quantified uncertainty is more accepted than consensus or vague statements (Gustafson and Rice, 2020; Kerr et al., 2023) but also that acceptance of the information depends on prior beliefs (Dries et al., 2025 and refs within) and that the impact is sensitive to receiver and format (Altenmüller et al., 2022; Stavrova et al., 2024; Van Der Bles et al., 2019). Despite calls to authors to embrace the inclusion of uncertainty to stay truthful (Burgio, 2019 and refs within), all the previous results serve to the reported hesitancy to visualize uncertainty to the public (Hullman, 2020).

Data uncertainty literacy is mostly studied and assessed as a task performing problem (Hullman et al., 2019). It means situations where a motivated user needs to evaluate specific information. For example, knowing when the bus will arrive, determining whether my location is hurricane safe, or assessing whether statistical differences exist.

## 3 The data visualization toolbox for uncertainty communication

Data visualization can help to easy task performance, that is, to improved accuracy and speed of the data understanding. It is a particularly difficult challenge with such a complex concept as uncertainty. This has been the focus of the work from the uncertainty visualization research community, a field that combines statistical knowledge, cognitive science, and visual design.

In technical or scientific heavy contexts, uncertainty is typically visualised with techniques from statistics like error bars, box plots, confidence intervals, or distribution functions. These are specialized visual devices that assume a certain level of statistical literacy. This default toolbox has been evaluated and advanced during last decades. The uncertainty visualization community has looked into the errors and reasons for misinterpretation and come up with best practices. Generic practical and theoretical standards are summarised in recent works (Franconeri et al., 2021; Hägele et al., 2022; Padilla et al., 2022; Padilla et al., 2021a), and paralleled with field-specific works like bioinformatics (Weiskopf, 2022), biological data targeted to decision makers (Levontin and Walton, 2020), meteorology (Calvo et al., 2022), and thesis (Hill, 2018; Yu, 2018). Following the lead of research, best practices have reached generic resources (e.g., Wilke, 2019).

The guiding criteria are reducing the user’s cognitive load and avoid them resorting to heuristics and subconscious mental shortcuts. This is achieved by minimizing the numeracy requirements (Batziakoudi et al., 2025) and ensuring congruence with salient features of information, effectively integrating understanding of uncertainty into pre-attentive processes (Padilla et al., 2021a).

## 4 Connect with the concept

Drawing on MacEachren’s semiotic framework, (MacEachren et al., 2005; 2012), a helpful distinction within the toolbox is between extrinsic signifiers, which represent uncertainty using additional geometric objects or marks (error bars, hatched overlays, glyphs, object contours), and intrinsic signifiers, where uncertainty is embedded within the visual channel of the data itself (desaturation, fuzziness/blur, transparency, texture, sketchiness). An example of this two approaches is the box plot vs. the density strip (Bowman, 2019).

This second family of techniques relies on instant cognitive processing through global, pre-attentive features. In doing so, it anchors uncertainty communication in the gist extraction phase of visual perception, when observers form an immediate impression of the overall scene (sensu Oliva and Torralba, 2006). This holistic first-pass perception can support the interpretation of visual metaphors by providing an immediate structural or semantic frame for comparison. In this sense, global properties map naturally onto visual metaphors to ensure that the viewer’s first impression aligns with the statistical reality of the data. This is how intrinsic approaches connect with the core idea and visual gist of what data uncertainty represents: doubt, lack of clarity, and weakened signal strength. It is helpful to think in term of signal (data) and noise (uncertainty), where “data value’s visual saliency is proportional to its certainty” (Feng et al., 2010; Mason et al., 2024). Signal suppression techniques support intuitive interaction and spontaneous interpretation because they embed cognitive affordance, that is, they lower the cognitive load to make sense of the data (Fygenson et al., 2025; Padilla et al., 2021b).

These methods have been evaluated for, e.g., biomolecular structures (Sterzik et al., 2025), borderization in maps (Libertini et al., 2021), and are the basis for novel approaches like visual entropy (Holliman et al., 2022), or value suppressing palettes (Correll et al., 2018).

The implementation of related data uncertainty visualization strategies is getting easier with specific codes developed to such effect. Recent examples are the Rpackage “ggdibbler” (Mason et al., 2026) (based on “distributional” by O’Hara-Wild et al. (2026)), which offers a flexible tool for uncertainty visualization that handles any plot, data, and distribution type. And the “bootplot” Phyton package (Petek et al., 2025) which generates hypothetical outcome plots and animations via bootstrap method of the original data.

## 5 Connect with the user

Addressing a non-expert audience that is not *a priori* motivated calls for visualization strategies that do not assume literacy, and also avoid the pervasive deterministic construal errors (Joslyn and Savelli, 2021). Such are the intrinsic, metaphor-laden intuitive visual representations of uncertainty (Section 4), where the visual attribute immediately communicates a statistical reality without requiring a user to read a legend or have prior statistical training.

In addition, if there is not *a priori* motivation, we need to engage the audience first, and capture their curiosity as a prelude to conveying the central message. A way of achieving this is by leveraging perceptual curiosity, that is, the drive to investigate novel or pleasant visual stimuli (as per Berlyne, 1954) as a door to epistemic curiosity (as per Loewenstein, 1994). This is akin to a “welcome gesture” concept, widely used, for example, in science journalism (Christiansen, 2023; Hatch and Jackson, 2022). It works as a narrative hook that transforms passive onlookers into information seekers.

**FIGURE 1:**
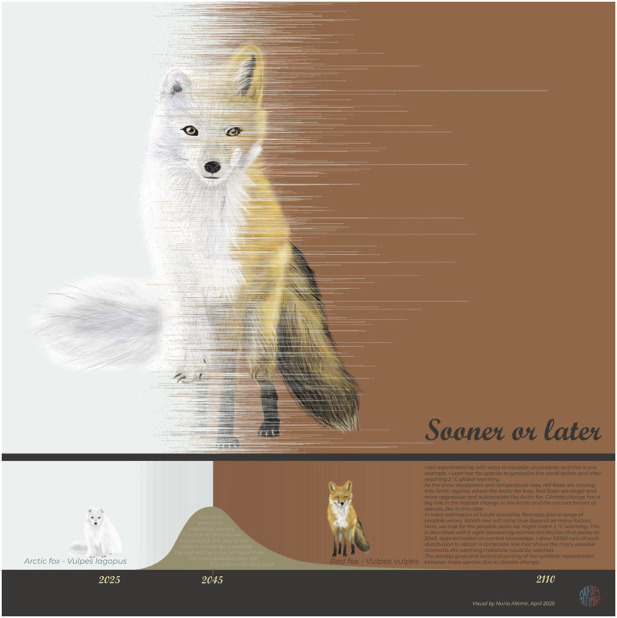
Example of data-driven visual infused with artistic expression. Image based on the submission to the VIZBI25 image contest. The overarching goal of that image is to highlight that model estimation of any event has a probability attached to it. The subject of choice for the submitted image was the forecast of the year when 2 °C global degree will be reached. The figurative parts of the illustration are blended with the computational output of the hypothetical outcomes. The illustration could have been any event, in this particular case, it displays the replacement of the Arctic fox by the Red fox. The irregular boundary is created with 10,000 hypothetical values sampled from the probability distribution function. The visual element that creates interests (the strange division between the two sides of the image) is also holds the gist of the information (the range of uncertainty).

The WWF Japan campaign on dwindling wildlife populations (Hakuhodo, 2008) illustrates a design choice that achieves both perceptual curiosity and immediate intuitive understanding, in this case, of conservation statistics. In their species portraits, the concept of disappearance is mapped to the destruction of visual resolution producing image blurriness. This blurriness was created via pixelation proportional to current population size, allowing viewers to grasp the severity of the decline at a glance. Another example is the work that prompted the writing of this piece, my own image presented to the VIZBI2025 image contest (Figure 1), where the visual element that creates interest also holds the core of the information.

These works aim to use aesthetic qualities, beauty, visual appeal, or sensory allure, to capture immediate attention and create an entry point for reflective cognitive processes, that should eventually lead to assimilation of new information. This mechanism is captured in the PIA-Pleasure-Interest-Model of Aesthetic Liking (Graf and Landwehr, 2015; Graf and Landwehr, 2017) by proposing a dual-process account in which aesthetic pleasure can arise from both fluent, effortless processing and from the interest generated by disfluency. Unexpected or complex stimuli can be rewarding when their ambiguity is resolved, satisfying curiosity and deepening understanding. These mechanisms can be framed in terms familiar to both cognitive science and the visual arts: expectations, predictions, and the brain’s drive to minimize prediction error. Predictive-processing accounts of aesthetics (Muth and Carbon, 2016; Van de Cruys and Wagemans, 2011; Van de Cruys et al., 2024; Frascaroli et al., 2024; Kesner, 2014) describe aesthetic pleasure as emerging from the reduction of prediction error, the “Aha!” moment when ambiguity resolves. This view aligns with broader evolutionary accounts of fitness-maximizing perception (Schaffner et al., 2023). It also resonates with artistic practice, where techniques that gently violate expectations can evoke curiosity or awe without aiming for the full spectrum of aesthetic emotions (Stamkou et al., 2024).

Taken together, these perspectives suggest that it should be possible to design data uncertainty visualizations that incorporate aesthetic qualities (richness, subtle complexity, or mild expectation-violation) to spark interest and encourage viewers to linger. Such designs can support sense-making by aligning with how viewers naturally resolve ambiguity, offering an experience that is both engaging and conducive to absorbing the underlying information. In doing so, the visualization supports an enjoyable experience that meets users where they are.

## 6 Discussion

The search for an aesthetic quality that “intuitively convey a sense of uncertainty” approaches us to the resources used in the fine arts (Hill et al., 2018). Art conveys uncertainty through ambiguity, complexity, coexisting meaning, expectation violation, or visibility of process hesitation. Intrinsic signifiers transmit uncertainty via ambiguous edges via e.g., blur or sketchiness. The example in Figure 1 transmits uncertainty using ambiguous spatial location of the date of interest and the texture it generates together with the illustrative elements.

The intentional incorporation of visual complexity would work against efficient processing of a data visualization, as mention in Section 3, because it introduces clutter, increases perceptual load, and slows rapid, accurate data interpretation (Leffrang and Müller, 2025). As discussed, this is especially true for motivated users who approach visualization with a specific task in mind, but as suggested in Section 5, it may be an asset for the engagement of non-expert users. Hullman et al. (2011) already described how ‘visual difficulties’ promote active processing and engagement and anchors the attention of a non-motivated user.

**FIGURE 2:**
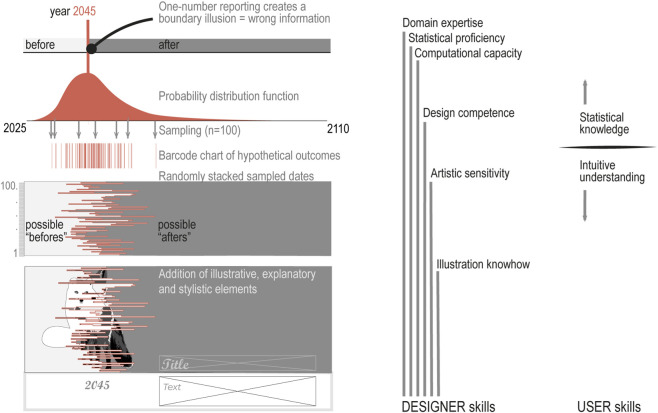
The elements and logics to construct Figure 1. The different expression of the date of interest (year when 2 °C global degree will be reached) is marked in red. The number of sampled outcomes in this example is 100 (vs. 10,000 in Figure 1). The image shows the same information in several ways: a classical area chart of the probability density function of the forecast, a barcode chart builds with hypothetical outcomes based on the function, and an illustration that portrays one consequence of global warming. The left hand side shows the skills expected from the designer and the user.

Complexity can also be conceptual, inherent to the information. We can choose to use complexity at any step of the process and, as Windhager et al. (2024) propose, we can view complexity as an element of information design. Taking again the example in Figure 1, visual complexity plays the interest arising role, and at the same time the conceptual complexity of uncertainty is preserved. This follows from the decision to connect with the gist and the refusal to simplify the information (Figure 2). The amount of ambiguity rendered corresponds with the actual estimated uncertainty range.

Being at the edge of artistical expression benefits from the visual and cognitive lure found in art. But being complex just for the sake of artistry is not the point, passing of knowledge is non-negotiable. Aligning with the cognitive models introduced in Section 5, complexity drives interest if resolvable, therefore we are aiming at a balance point. A balance between order and complexity is important for aesthetic appreciation (Van Geert and Wagemans, 2020). Also, whether a data visualization will be remembered as image or information depends on the relative amount of graphical elements (Arunkumar et al., 2025; 2023). Caution is also advised regarding the addition of relatable illustrations intended as a welcoming anchor (the foxes in Figure 1). As (Madan et al., 2018) shows emotional arousing visuals elevate the perception of complexity of images.

I have argued that intuitive encoding and aesthetic lure offer accessible ways to communicate data uncertainty to non-expert audiences. By removing technical and cognitive demands, they are suited to become default visualization practice. That alone, making data uncertainty visible, would advance uncertainty literacy, and repeated exposure would increase familiarity, preference, and visual comfort, gradually normalizing data uncertainty and its visualization.

For this practice to be widespread, statistical, design, and art understanding should overlap. This is not always the case. Data visualization exists at the crossroads of multiple disciplines, each with its own norms, incentives, and literacies, conditions that can foster siloed practices. At the extremes, scientists do not incorporate artistical creativity and design thinking, and designers are not statistically literate. This is akin to the underlying tension between statistical graphics and information visualization discussed in the exchange between Gelman and Unwin (2013) and Kosara (2013). As also generically discussed in (Ghisleni et al., 2025) for the art-science intersection. The disciplinary gap has been implicitly or explicitly discussed in the field of uncertainty visualization, as in Mason et al. (2024) discussing the disconnect where statisticians and designers do not share a common language.

Discipline disconnect appears at many levels. For example, Lan and Liu (2025) show, the designer tendency to outweigh beauty over science and/or lack or data literacy are behind some of the design flaws produced by “everyday designers”. And Branson et al. (2025) discuss the disconnect in college-level data visualization courses, with a survey that shows that courses focus on design topics, whereas very few touch on statistical principles. Emphasis on the need for interdisciplinary collaboration is also voiced in Battisti and Daquino (2025), who report on the existing gap in digital humanity projects, which rarely implement uncertainty-aware visualization designs.

Overall, these insights point to the need for a more integrated practice, one in which statistical rigor, design literacy, and appreciation for aesthetic are mutually reinforcing components of effective communication (Figure 2). Data uncertainty visualization, in particular, stands to benefit from this convergence: it requires the precision of science, the sensitivity of design, and the interpretive openness often found in art. Moving toward such a hybrid mindset would enable visualizations that are both intellectually honest and experientially engaging, helping audiences become more comfortable with uncertainty, more attentive to nuance, and better equipped to learn from the data that shape our world.

## Data Availability

The raw data supporting the conclusions of this article will be made available by the authors, without undue reservation.
